# A case of acute Sheehan’s syndrome and literature review: a rare but life-threatening complication of postpartum hemorrhage

**DOI:** 10.1186/s12884-017-1380-y

**Published:** 2017-06-14

**Authors:** Shinya Matsuzaki, Masayuki Endo, Yutaka Ueda, Kazuya Mimura, Aiko Kakigano, Tomomi Egawa-Takata, Keiichi Kumasawa, Kiyoshi Yoshino, Tadashi Kimura

**Affiliations:** Department of Obstetrics and Gynecology, Osaka University Graduate School of Medicine, 2-2 Yamadaoka, Suita, Osaka, 565-0871 Japan

**Keywords:** Hyponatremia, Hypopituitarism, Postpartum hemorrhage, Sheehan syndrome, Sheehan’s syndrome, Subsequent pregnancy

## Abstract

**Background:**

Sheehan’s syndrome occurs because of severe postpartum hemorrhage causing ischemic pituitary necrosis. Sheehan’s syndrome is a well-known condition that is generally diagnosed several years postpartum. However, acute Sheehan’s syndrome is rare, and clinicians have little exposure to it. It can be life-threatening. There have been no reviews of acute Sheehan’s syndrome and no reports of successful pregnancies after acute Sheehan’s syndrome. We present such a case, and to understand this rare condition, we have reviewed and discussed the literature pertaining to it. An electronic search for acute Sheehan’s syndrome in the literature from January 1990 and May 2014 was performed.

**Case presentation:**

A 27-year-old woman had massive postpartum hemorrhage (approximately 5000 mL) at her first delivery due to atonic bleeding. She was transfused and treated with uterine embolization, which successfully stopped the bleeding. The postpartum period was uncomplicated through day 7 following the hemorrhage. However, on day 8, the patient had sudden onset of seizures and subsequently became comatose. Laboratory results revealed hypothyroidism, hypoglycemia, hypoprolactinemia, and adrenal insufficiency. Thus, the patient was diagnosed with acute Sheehan’s syndrome. Following treatment with thyroxine and hydrocortisone, her condition improved, and she was discharged on day 24.

Her next pregnancy was established 2 years after her first delivery. She required induction of ovulation for the next conception. The pregnancy, delivery, and postpartum period were uneventful. An electronic search of the literature yielded 21 cases of acute Sheehan’s syndrome. Presenting signs varied, including adrenal insufficiency (12 cases), diabetes insipidus (4 cases), hypothyroidism (2 cases), and panhypopituitarism (3 cases), with a median time of presentation after delivery for each of those conditions being 7.9, 4, 18, and 9 days, respectively. Serial changes in magnetic resonance imaging were reported in some cases of acute Sheehan’s syndrome.

**Conclusion:**

Clinicians should be aware of the risk of acute Sheehan’s syndrome after a massive postpartum hemorrhage in order to diagnose it accurately and treat it promptly.

## Background

Postpartum hemorrhage (PPH) is an obstetric emergency that occurs in 1–2% of live births [1]. Sheehan’s syndrome is well known as a complication of PPH; this condition can present with chronic symptoms after a relatively long latent period, including failure to lactate, mild headache, fatigue, nausea, and amenorrhea [2]. Although the frequency of Sheehan’s syndrome has decreased because of recent advances in obstetrical care, Sheehan’s syndrome is one of the most important causes of hypopituitarism [3]. Sheehan’s syndrome is generally diagnosed several years postpartum; therefore, it has been recognized as a chronic condition. However, in some cases, Sheehan’s syndrome presents with acute symptoms and this variant may be life threatening [3]. Here we report our experience with a case of this rare type of Sheehan’s syndrome and present a literature review of this condition.

## Case presentation

A 27-year-old woman (gravida 1, para 0) delivered a healthy 2715-g female infant at 40 weeks of gestation in a private hospital. The patient had no notable medical or family history and had not experienced problems during the course of her pregnancy. She developed a massive hemorrhage at the time of delivery and transferred to our hospital for emergency treatment. The estimated total blood loss was approximately 4000 mL on admission.

On admission to our hospital, the patient had a blood pressure of 110/50 mmHg and a heart rate of 140 beats/min. A complete blood count (CBC) revealed a hematocrit (HCT) level of 11.4% and a hemoglobin (Hb) level of 4.1 g/dL. We immediately started transfusion and uterotonic therapies and employed uterine embolization, which successfully stopped the bleeding. We estimated her total blood loss to be approximately 5000 mL at this point, and she received 3500 mL of fluid infusions, including 2800 mL of red blood cells, 1200 mL of fresh frozen plasma, and 400 mL of platelets by transfusion. Subsequently, her CBC recovered (HCT: 27.6% and Hb: 9.6 g/dL) and vital signs stabilized (blood pressure: 140/90 mmHg and heart rate: 100 beats/min).

On day 7 postpartum, the patient’s general condition was relatively stable, with no evidence of hypotension, general fatigue, headache, failure to lactate, or hypoglycemia; therefore, we planned to discharge her on the following day. However, on day 8, the patient experienced a grand mal seizure and became comatose. Because her oxygen saturation level decreased to 60%, she was sedated and intubated. The laboratory data showed slightly low Hb (10.3 g/dL) and HCT levels (29.7%), a normal platelet count (262 × 10^3^/μL), and low sodium (Na^+^, 111 mEq/L) and chloride (Cl^−^, 84 mEq/L) levels. Other tests were within the normal range. Magnetic resonance imaging (MRI) of the pituitary gland revealed no abnormalities and intracranial hemorrhage could be ruled out. Consequently, we diagnosed that her seizure was caused by hyponatremia and started the appropriate NaCl replacement treatment to increase her Na^+^ levels by 10 mEq/day.

**Fig. 1:**
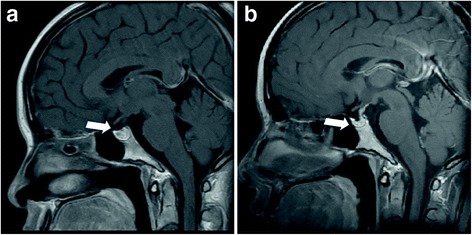
Gadolinium-enhanced T1-weighted magnetic resonance image of the pituitary gland. **a** No change was observed in the pituitary gland on postpartum day 15 (day 7 after the seizure). **b** Marked diminution in the pituitary gland size was observed after 6 months. The *white arrow* indicates the pituitary gland

The patient’s hormone levels on postpartum day 8 are shown in Table 1a. These results suggested that the pituitary dysfunction was either due to Sheehan’s syndrome or lymphocytic hypophysitis. The adrenal insufficiency was treated with hydrocortisone. A second MRI scan did not reveal lymphocytic hypophysitis, and a Gadolinium-enhanced T1-weighted MRI showed a normal pituitary gland on postpartum day 15 (7 days after the seizure; Fig. 1a). On day 17, after the seizure, we performed hormone stimulation tests for corticotropin-releasing hormone (CRH), luteinizing hormone-releasing hormone (LH-RH), and thyrotropin-releasing hormone (TRH) and on day 18, we performed a stimulation test for growth hormone-releasing peptide (GH-RP). The hormone levels in Table 1b demonstrated low reactivity for ACTH, TSH, FSH, prolactin (PRL), and growth hormone (GH). Based on these results, we diagnosed the patient with acute Sheehan’s syndrome. Following treatment with thyroxine and hydrocortisone, her condition improved.

**Table 1:** (a) The results of hormone levels at the day of seizure. The pituitary dysfunction was observed. (b) The results of CRH, TRH, LH-RH, and GH-RP stimulation test. Low reactivity for ACTH, TSH, FSH, prolactin (PRL), and growth hormone (GH) was observed

a							
The day after seizure		Day 0					
	normal range						
adrenocorticotropic hormone (ACTH)	0–60 pg/mL	14					
cortisol	4.3–20 μg/dl	3.4					
thyroid-stimulating hormone (TSH)	0.400–4.80 MU/mL	0.72					
free thyroxine	0.80–1.90 ng/dL	0.9					
PRL	3.50–30.00 ng/mL	11.3					
growth hormone (GH)	0.1–2.7 ng/mL	0.18					
b							
The day after seizure		Day 17			6 months		
duration after the stimulation		0 min	30 min	60 min	0 min	30 min	60 min
	normal range	none	CRH stimulation	CRH stimulation	none	CRH stimulation	CRH stimulation
adrenocorticotropic hormone (ACTH)	0–60 pg/mL	9	19	18	<5.0	12	20
		none	TRH stiumulation	TRH stiumulation	none	TRH stiumulation	TRH stiumulation
thyroid-stimulating hormone (TSH)	0.400–4.80 MU/mL	0.69	4.32	2.98	0.75	4.62	3.16
		none	LH-RH stiumulation	LH-RH stiumulation	none	LH-RH stiumulation	LH-RH stiumulation
FSH	0.4–11 mIU/ml	7.5	9.9	10.5	1.2	4.2	4.6
LH	0.08–7.3 mIU/mL	1.6	3.3	3.5	3.1	8.3	9.7
		none	TRH stiumulation	TRH stiumulation	none	TRH stiumulation	TRH stiumulation
PRL	3.50–30.00 ng/mL	10.9	20.9	16.9	10	32.6	22.5
		none	GH-RP stiumulation	GH-RP stiumulation	none	GH-RP stiumulation	GH-RP stiumulation
growth hormone (GH)	0.1–2.7 ng/mL	0.21	3.05	1.91	0.07	2.35	0.75

*Abbreviations: ACTH* adrenocorticotropic hormone, *CRH* corticotropin-releasing hormone, *FSH* follicle-stimulating hormone, *GH* growth hormone, *GH-RP* growth hormone releasing peptide, *LH-RH* luteinizing hormone-releasing hormone, *PRL* prolactin; *TRH* thyrotropin-releasing hormone, *TSH*, thyroid-stimulating hormone

We discharged the patient on day 24 postpartum and advised her to continue the hydrocortisone 15 mg/day and thyroxine sodium 75 mg/day treatment. At 6 months postpartum, the follow-up CRH-, LH-RH-, and TRH-stimulation tests revealed persistent pituitary dysfunction (Table 1b), and we diagnosed her with generalized hypopituitarism. A repeat MRI at this point also revealed an atrophic pituitary gland (Fig. 1b) without clinical evidence of neurological impairment. At 12 months postpartum, she developed a pituitary amenorrhea that necessitated estrogen and progesterone hormone treatment. Her next pregnancy was established after two years of her first delivery using ovulation-inducing hormones and human menopausal gonadotropin–human chorionic gonadotropin (hMG-hCG) treatment. During her pregnancy, she was maintained on hydrocortisone 15 mg/day and thyroxine sodium 75 mg/day. Fetal growth was determined to be appropriate for gestational age, and no major anomalies were observed on ultrasound at 19, 28, 32, 36, and 40 weeks of gestation. At 41 weeks of gestation, she required labor induction and delivered a healthy 2870-g female infant with Apgar scores of 8 and 9 at 1 and 5 min, respectively. A total amount of 100 mg of hydrocortisone was added as a steroid cover at the time of delivery. There were no complications postpartum and she was discharged 5 days postpartum. At 12 months after her second delivery, amenorrhea was also observed and necessitated repeat estrogen and progesterone hormone treatment.

## Discussion

Sheehan’s syndrome was first described by Sheehan in 1937 [4]; through improved management of hemodynamic complications, its incidence has gradually declined over time. Although the exact incidence is unknown and it rarely occurs in modern obstetric practices, Sheehan’s syndrome still must be considered in cases of PPH. Sheehan’s syndrome is pituitary necrosis after PPH and hypovolemia and occurs in 1–2% of women who lose 1–2 L of blood with associated hypotension [5, 6]. Several studies have shown that the latent period between symptoms and postpartum hemorrhage can be several years in Sheehan’s syndrome [2, 4, 7]. For example, in 1999, Banzal et al. reported significant delays between symptom onset and postpartum hemorrhage, with only two patients diagnosed within a year, 20 patients that were symptomatic for over 6 years before diagnosis, and appropriate treatment [7]. Symptoms that first occur within 6 weeks postpartum are defined as acute Sheehan’s syndrome in this report because the postpartum period is commonly identified as 6 weeks after delivery.

Our case presented with a life-threatening seizure and coma as the first symptoms. Our literature review revealed that such marked and abrupt onset in Sheehan’s syndrome are rare. The primary causes of postpartum seizures include eclampsia, cerebral ischemic changes, cerebral hemorrhage, cerebral venous sinus thrombosis, reversible cerebral vasoconstriction syndrome, epilepsy, and hypoglycemia [8]. None of the imaging results in our case revealed any brain disorders, and because the laboratory investigations revealed hyponatremia and following low hormonal levels, we reasonably concluded that our final diagnosis was acute Sheehan’s syndrome with the loss of general pituitary function.

Only a few studies have discussed acute Sheehan’s syndrome, and we found no literature review on this topic. Therefore, we performed a PubMed/Medline and Google Scholar search of the English literature between January 1990 and May 2014, using the key words “acute” or “early” and “Sheehan syndrome” or “Sheehan’s syndrome.” Searching with the keywords mentioned above, 52 articles were identified. We read these articles and selected those cases with patients whose symptoms first occurred within 6 weeks postpartum; this identified 19 suitable reports (21 cases including our case). Nineteen full texts of articles were read by the authors. Twenty cases of acute Sheehan’s syndrome were published in international peer-reviewed literature. The 21 cases are summarized in Table 2 [9–27]. We selected the topics, which are distinct features of acute Sheehan’s syndrome for further discussion.

**Table 2:** A summary of the literature review findings for acute Sheehan syndrome

First author	Year (Reference number)	Age (years)	Day	cause of hemorrhage		Blood loss	Hb level	Shock	DIC	Symptom	Headache	Etiology	Treatment
Putterman C	1991 [9]	27	7 days	uterine atony	vaginal delivery	estimated blood loss was 2 L	not described	presented	not described	paresthesia	not described	hyponatremia, adrenal insufficiency	hydrocortisone, levothyroxine, estrogen and progesterone
The patient was resuscitated with blood and colloids; hemorrhage was controlled with uterine massage, oxytocin, and ergotamine.													
Syndrome of inappropriate secretion of antidiuretic hormone caused by Sheehan’s syndrome should be considered in the differential diagnosis of postpartum hyponatremia.													
Zuker N	1995 [10]	20	14 h	uterine atony	vaginal delivery	1200 mL	Decreased to 5.1 g/dl	presented	presented	hypoglycemia	not described	adrenal insufficiency	hydrocortisone.
An urgent subtotal hysterectomy was performed due to life threatening hemorrhage.													
Acute hypoglycemic coma as the initial manifestation of Sheehan’s syndrome in the first few hours postpartum is extremely rare.													
Lavallee G	1995 [11]	30	6 h	uterine inversion	vaginal delivery	not described	Decreased to 7.6 g/dl	not presented	not described	generalized tonic-clonic convulsions	presented	adrenal insufficiency	hydrocortisone and levothyroxine
She underwent a uterine revision under 5 mg intravenous midazolam hydrochloride.													
MR was performed 6 days after delivery; a large intrasellar mass with superior extension was confirmed on T1-weighted. The mass effect has disappeared, and the pituitary gland is somewhat atrophic (postpartum day 48)													
It is therefore important to be alert to the possibility that an enlarged nonhemorrhagic pituitary gland may be present in the post-infarction phase of Sheehan’s syndrome, as shown in the present case report													
Kan AK	1998 [12]	32	24 h	unclear	cesarean section	500 mL	Decreased to 5.7 g/dl	not presented	not described	excessive urination	not described	diabetes insipidus	desmopressin
She was transfused 4 units of blood and additional treatment was not performed.													
This is a report of a case of diabetes insipidus developing within 24 h postpartum in a grand multipara who had an elective lower segment Cesarean section for twins.													
Dejager S	1998 [13]	32	3 days	*	not described	little	not described	presented	not described	Severe headache excessive urination	presented	diabetes insipidus	hydrocortisone and desmopressin
The delivery was complicated by a occurrence of a severe hypotention episode at the beginning of the epidural anesthesia.													
MRI was performed 6 days after delivery. MRI revealed the presence of a holosellar 11-mm diameter mass.													
Follow-up MRI showed a spontaneous and rapid shrinkage of the pituitary, within 20 days, which appeared as an empty sella 3 months later.													
Sheehan’s syndrome may initially closely mimic hypophysitis, or the necrosis of an adenoma.													
Boulanger E	1999 [14]	30	10 days	uterine scar disjunction	VBAC	not described	not described	not described	presented	asthenia	not described	hyponatremia, adrenal insufficiency	glucocorticoids.
Hysterectomy was performed to control blood loss and transient disseminated intravascular coagulation occurred.													
MRI was not performed.													
The report of early and acute hyponatremia with inappropriate secretion of antidiuretic hormone occurring 10 days after vaginal delivery with severe blood loss.													
Kale K	1999 [15]	23	20 days	not described	not described	not described	not described	not described	not described	psychosis	not described	maybe hypothyroidism	predonisolone and thyroxine sodium
The treatment to control the bleeding was not described.													
MRI was not performed.													
It was interesting to note that all the clinical features of Sheehan’s syndrome and psychosis improved with hormone replacement therapy and she did not require treatment with antipsychotic medications.													
Schrager S	2001 [16]	39	12 days	atonic bleeding	cesarean delivery	severe	not described	presented	feeling nausea	general fatigue	not described	hyponatremia, adrenal insufficiency	cortisone acetate
Hysterectomy was performed to control blood loss and underwent an embolization of her right vaginal artery.													
A sodium level measured on the 5th day of her hospitalization was normal.													
Although Sheehan’s syndrome is uncommon as a result of improved obstetric care, it should be a consideration in any woman who has a history of a postpartum hemorrhage and who reports signs or symptoms of pituitary deficiency.													
Lust K	2001 [17]	32	3 days	atonic bleeding	vaginal delivery	3200 ml	not described	presented	presented	headache	presented	hyponatremia	thyroxine and cortisone acetate
Uterotonic agents successfully controled the bleeding.													
MRI scan of pituitary day five after delivery showed the enlarged pituitary gland with its superior margin reaching the undersurface of the optic chiasm.													
MRI scan of pituitary 4 months after delivery showed atrophic pituitary gland and empty sella.													
Wang HY	2002 [18]	32	7 days	persistent bleeding from uterus	cesarean delivery	severe	3.5 g/dL	presented	presented	excessive urination	not described	diabetes insipidus	desmopressin
Angiography with bilateral uterine artery embolization was performed.													
MRI was not performed.													
There are very few existing literature discussing concomitant Sheehan’s syndrome and acute renal failure.													
Bunch TJ	2002 [19]	23	6 days	atonic bleeding	cesarean delivery	massive hemorrhage	not described	presented	presented	general fatigue	not described	hyponatremia, adrenal insufficiency	predonisolone and levothyroxine
She received large volumes of fluid and blood products for resuscitation. Additional treatment was not performed.													
MRI demonstrates an enlarged pituitary gland with abnormal signal on the T1 weighted precontrast images (postpartum approximately day 10).													
There are many studies describing complications of late Sheehan’s syndrome; however, relatively few contain descriptions of the acute phase.													
Munz W	2004 [20]	33	6 days			Hb level decreased to 3.0 g/dL				headache, vomitting		hyponatremia, adrenal insufficiency	hydrocortisone and levothyroxine
Hysterectomy was performed to control blood loss. The patient received a transfusion of 12 units of blood and six units of fresh frozen plasma.													
MRI of the pituitary was normal on postpartum day 6.													
Sheehan’s syndrome can be associated with hyponatremia, illustrating the need to include hyponatremia as an initial symptom in the differential diagnosis of Sheehan’s syndrome.													
Wang S	2005 [21]	33	19 days	postpartum hemorrhage	cesarean delivery	Massive bleeding	6.6 g/dL	presented	presented	hemodynamic instability	not described	adrenal insufficiency	hydrocortisone and thyroxine sodium
Hysterectomy was performed to control blood loss.													
MRI showed no notable abnormality (postpartum day 19).													
MRI showed a flattened pituitary gland and loculation of cerebrospinal fluid (postpartum day 32).													
Although the occurrence of Sheehan’s syndrome is now rare, it should still be considered in any woman with a history of peripartum hemorrhage who develops manifestations of pituitary hormone deficiency.													
Kaplun J	2008 [22]	29	17 days	retained placenta	unknown	massive	3.8 g/dL	not described	not described	general fatigue	presented	panhypopituitarism	not described
		21	3 days	perineal laceration	vaginal delivery	massive	5.5 g/dL	presented	not described	fever and a severe headache	not presented	hyponatremia, adrenal insufficiency	prednisone and levothyroxine
The treatment used to control the bleeding was not described for either case 1 or 2.													
MRI on postpartum day 26 revealed a nonenhancing, minimally hypointense lesion in the pituitary gland (case 1).													
MRI obtained on postpartum day 6 showed an enlarged pituitary gland with suprasellar extension to the optic chiasm (case 2).													
Anfuso S	2009 [23]	35	8 days	none	vaginal delivery	500 mL	8.8 g/dL	not presented	not presented	asthenia, persistent headache	not presented	hyponatremia, adrenal insufficiency	hydrocortisone and levothyroxine
The treatment to control the bleeding was not described.													
MRI on postpartum day 8 revealed an abnormal lack of enhancement of pituitary grand.													
MRI 3 months postpartum confirmed previous vascular necrosis.													
Early diagnosis of early-onset Sheehan’s syndrome associated with severe hyponatremia, following dystocic childbirth complicated by postpartum hemorrhage.													
Kumar S	2011 [24]	36	4 days	atonic bleeding	vaginal delivery	massive	6.1 g/dL	presented	presented	excessive urination	not presented	diabetes insipidus	desmopressin
Hysterectomy was performed to control the blood loss. The patient received the massive transfusion.													
MRI showed a normal pituitary gland (postpartum day 6).													
It is important to consider posterior pituitary ischemia resulting from Sheehan’s syndrome, presenting as central diabetes insipidus, as a cause of polyuria. Appropriate hormonal replacement that is initiated early can improve the clinical status and outcomes of patients.													
Shoib S	2013 [25]	31	16–18 days	unknown	unknown	not described	not described	not described	not described	psychosis	not described	possibly hypothyroidism	prednisolone and thyroxine sodium
The treatment to control the bleeding was not described.													
CT and MRI scans were not performed.													
Psychosis in patients with Sheehan’s syndrome is uncommon. Clinicians should have a high index of suspicion when postpartum-psychosis presents with a significant obstetric history.													
Sasaki S	2014 [26]	37	4–6 days	retained placenta	vaginal delivery	massive	4.0 g/dL	presented	presented	failure to lactate	not described	panhypopituitarism	hydrocortisone.
Emergency uterine embolization was performed.													
Sagittal T1-weighted image showing slight swelling of the anterior lobe and the pituitary stalk (postpartum day 10).													
At 1 month after delivery, swelling of the anterior lobe was reversed.													
At 5 months after delivery, marked atrophy of the anterior lobe was observed													
Hale B	2014 [27]	31	6 days	retained placenta	vaginal delivery	1500 ml	6.2 g/dL	presented	presented	headache, failure to lactate, fatigue	presented	partial hypopituitarism	prednisone, levothyroxine, desmopressin and somatropin
Retained placenta required manual extraction.													
Cranial magnetic resonance imaging scan performed on postpartum day six. The pituitary gland appears enlarged with peripheral enhancement and an isodense central area.													
Postpartum headache is a common occurrence with a broad differential diagnosis. Combined pathophysiological features of Sheehan’s syndrome and postpartum headache is an atypical acute presentation.													
Present case	2015	27	8 days	atonic bleeding	vaginal delivery	at least 5000 mL	4.1 g/dl	presented	presented	grand mal convulsion	not presented	hyponatremia, adrenal insufficiency	hydrocortisone and thyroxine sodium
Emergency uterine embolization was performed to control the blood loss.													
A sagittal T1-weighted image of the pituitary gland was normal on postpartum day 15.													
At 6 months after delivery, marked atrophy of the anterior lobe was observed.													
Early onset of Sheehan’s syndrome is rare. Acute Sheehan’s syndrome presenting with a sudden onset of postpartum seizures is rarer still.													
VBAC: Vaginal birth after cesarean section													

*Severe hypotension episode at the beginning of the epidural anesthesia with loss of consciousness was observed

*Abbreviations: CT* computed tomography, *Hb* hemoglobin, *MRI* magnetic resonance imaging

Of the identified cases, 17 described the amount of postpartum bleeding. Most patients suffered a massive hemorrhage; however, in one case, a PPH of approximately 500 mL caused acute Sheehan’s syndrome [23]. These results suggested that most cases of acute Sheehan’s syndrome occurred after PPH, which is well known for traditional Sheehan’s syndrome.

The Hb levels were mentioned in 12 cases, all of which experienced a moderate to severe decrease in Hb (approximately 3.0–8.8 g/dL). These results suggested that anemia is a reason for acute Sheehan’s syndrome. However, some cases were not associated with either PPH or anemia.

It is difficult to discuss the relationship between hypotension and acute Sheehan’s syndrome because the definition of shock was unclear in the previous reports. However, 12 of the 21 cases had experienced hypotension below 90 mmHg, which suggested that hypotension is a risk factor for acute Sheehan’s syndrome. Dejager et al. reported an interesting case showing severe hypotension because of an epidural anesthesia causing acute Sheehan’s syndrome; this study revealed that only hypotension may be cause the acute Sheehan’s syndrome [13].

PPH, anemia, and hypotension may decrease the blood flow to the pituitary and lead to necrosis of the gland. To prevent acute Sheehan’s syndrome, the obstetrician should strive to prevent anemia and hypotension in the treatment of PPH.

The first signs were reported within 3 days, 4–10 days, and 11–20 days postpartum in 6, 10, and 5 cases, respectively. Sixteen of 21 patients experienced the first signs within 10 days postpartum. Therefore, the obstetrician should be particularly vigilant for this condition during this time period. The first signs were hyponatremia because of adrenal insufficiency in 12 cases, diabetes insipidus in 4, hypothyroidism in 2, and panhypopituitarism in 3. Notably, the length of time after delivery until the first sign was noted varied depending on the cause. Adrenal insufficiency presented at a median of 7.9 days (14 h–19 days), diabetes insipidus at 4 days (1–7 days), hypothyroidism at 18 days (16–20 days), and panhypopituitarism at 9 days (4–17 days). Although the reasons for postpartum headache vary and may be difficult to diagnose accurately, acute Sheehan’s syndrome is one possible cause. In our literature review, 6 of 21 patients reported severe headache on the day of delivery. There was no correlation between the presence of headache and the onset of signs indicating acute Sheehan’s syndrome. The presence of severe headache on the day of delivery may indicate intracranial hemorrhage, but if that can be ruled out, the obstetrician should remain alert for the possible onset of acute Sheehan’s syndrome.

MRI evidence of acute Sheehan’s syndrome was reported in 13 cases. Early radiologic findings within postpartum day 20 were reported in 11 cases. In our case, the MRI findings were normal on postpartum day 15 (7 days after the seizure). Two studies reported that the MRI scans were normal on postpartum day 6; in addition, another study reported that normal scans were obtained on day 19 [20, 21, 24]. Seven studies reported their early MRI findings [11, 13, 17, 19, 22, 23, 26]. Lavallee et al. and Dejager et al. reported a large intrasellar mass with superior extension was confirmed on T1-weighted on day 6 [11, 13]. Bunch et al., Kaplun et al. and Sasaki et al. reported an enlarged pituitary gland with an abnormal signal on the T1-weighted pre-contrast images on postpartum day 10, day 10 and day 6, respectively [19, 22, 26]. Anfuso et al. described MRI findings from postpartum day 8 that showed an abnormal lack of pituitary gland enhancement [23]. Although the data was limited, in four of eleven cases, there were insignificant findings because of acute Sheehan’s syndrome on the MRI within postpartum 20 days. A large intrasellar mass with superior extension, an enlarged pituitary gland with an abnormal signal and an abnormal lack of pituitary gland enhancement were reported as early radiologic findings of acute Sheehan’s syndrome.

Including our case, marked findings associated with Sheehan’s syndrome have been observed at day 26, day 32, 3 months, 5 months, and 6 months [18, 22, 23, 26]. Specific findings included a non-enhancing, minimally hypointense lesion in the pituitary gland on day 26 postpartum [22] and a hypointense area with a flattened pituitary gland on day 32 postpartum [21]. An empty sella was observed several months postpartum [13, 22, 23, 26].

Pregnancy after acute Sheehan’s syndrome has not been reported. Whether the patient needs an induction of ovulation or not depends on the severity of this condition. If the patient did not have menstrual cycles because of hypopituitarism, the patient may need an induction of ovulation to establish a pregnancy. Our patient had no menstrual cycles; therefore, the patient received hMG-hCG treatment and established a pregnancy.

A study of a large number of pregnancies in hypopituitarism has not been reported. The largest number of cases was reported by Kübler et al. who reviewed pregnancy management in women with hypopituitarism [28]. Based on the analysis of 31 pregnancies in 27 women, they concluded that women with hypopituitarism were at an increased risk of obstetrical complications; postpartum hemorrhage occurred in 8.7%, transverse lie occurred in 16, and 42.4% of the newborns who were small for gestational age. Fortunately, our patient had no obstetrical complications other than the postdate delivery.

The postdate delivery was probably not because of the loss of general pituitary function because a review reported no increase in the rate of postdate deliveries in women with hypopituitarism [28]. In addition, animal data has shown that the delivery proceeded normally in oxytocin-deficient mice [29]. However, because there have been few reported cases of pregnancy with generalized hypopituitarism, many such cases will be need to be studied to obtain more human data.

## Conclusion

In summary, we report a rare case of Sheehan’s syndrome in a woman with early symptoms of life-threatening seizures, coma, and respiratory failure. After an initial resuscitation and treatment for PPH, the symptoms of Sheehan’s syndrome significantly improved with appropriate hormone replacement treatment. Although healthcare providers should be aware of the possibility of Sheehan’s syndrome that occurs several years postpartum complicated by PPH, they should also consider the possibility of acute presentations of Sheehan’s syndrome.

## Abbreviations

ACTH: Adrenocorticotropic hormone
CRH: Corticotropin-releasing hormone
FSH: Follicle-stimulating hormone
GH: Growth hormone
GH-RP: Growth hormone-releasing peptide
Hb: Hemoglobin
HCT: Hematocrit
LH-RH: Luteinizing hormone-releasing hormone
MRI: Magnetic resonance imaging
NaCl: Sodium chloride
PPH: Postpartum hemorrhage
PRL: Prolactin
TRH: Thyrotropin-releasing hormone
TSH: Thyroid-stimulating hormone
